# Helical and skyrmion lattice phases in three-dimensional chiral magnets: Effect of anisotropic interactions

**DOI:** 10.1038/s41598-017-07907-0

**Published:** 2017-08-07

**Authors:** J. Chen, W. P. Cai, M. H. Qin, S. Dong, X. B. Lu, X. S. Gao, J.-M. Liu

**Affiliations:** 1 Institute for Advanced Materials, South China Academy of Advanced Optoelectronics and Guangdong Provincial Key Laboratory of Quantum Engineering and Quantum Materials, South China Normal University, Guangzhou, 510006 China; 2 Department of Physics, Southeast University, Nanjing, 211189 China; 3 Laboratory of Solid State Microstructures, Nanjing University, Nanjing, 210093 China

## Abstract

In this work, we study the magnetic orders of a classical spin model with anisotropic exchanges and Dzyaloshinskii-Moriya interactions in order to understand the uniaxial stress effect in chiral magnets such as MnSi. Variational zero temperature calculations demonstrate that various helical orders can be developed depending on the interaction anisotropy magnitude, consistent with experimental observations at low temperatures. Furthermore, the uniaxial stress induced creation and annihilation of skyrmions can be also qualitatively reproduced in our Monte Carlo simulations. Our work suggests that the interaction anisotropy tuned by applied uniaxial stress may play an essential role in modulating the magnetic orders in strained chiral magnets.

## Introduction

In the past years, the nontrivial magnetic orders observed in chiral magnets such as MnSi^1–3^, Fe_1−*x*_Co_*x*_Si^4^ and FeGe^5, 6^ have been attracting continuous attention due to the interesting physics and potential applications for future memory technology. Specifically, a helical order with a single ordering wave vector **k** (point along the[111] axis in MnSi, for example) is developed at low temperatures (*T*) under zero magnetic field (*h*), resulting from the competition between the ferromagnetic (FM) exchange interactions and the Dzyaloshinskii-Moriya (DM) interactions^7, 8^. When a finite *h* is applied, the helical order is replaced by a conical phase to save the Zeeman energy. More interestingly, a skyrmion lattice phase^9^ (a vortex-like spin configuration where the spins projected on a sphere point radially) is stabilized in a certain (*T*, *h*) region, and is proposed to be potentially used for data encoding because of its efficient modulation by ultralow current density^10, 11^ (~10^5^–10^6^ A m^−2^, orders of magnitude smaller than that for domain-wall manipulation) and its topological stability. Theoretically, the cooperation of the energy competition (among the FM, DM, and Zeeman couplings) and thermal fluctuations is suggested to stabilize the skyrmion lattice phase^12^ in bulk chiral magnets, and the Rashba spin-orbit coupling in two-dimensional materials is believed to further enhance the stability of skyrmions^13^.

Subsequently, a number of theoretical simulations searching for effective methods for manipulating skyrmions have been performed in order to develop related spintronic devices. It is suggested that skyrmions in bulk and/or thin film systems could be controlled by external stimuli such as electric currents^14^, magnetic fields^15^, and thermal gradients^16, 17^. As a matter of fact, some of these manipulations have been experimentally confirmed^18^, although it is very hard to create and annihilate skyrmions using these methods^19, 20^.

Most recently, the dependence of magnetic orders on uniaxial stress in MnSi has been investigated experimentally in details^21, 22^. The wave vector of the helical order at zero *h* is reoriented from the111 axis to the stress axis when a uniaxial stress is applied. More importantly, the *T*-region of the skyrmion lattice phase can be extensively modulated by stress, demonstrating an additional method to manipulate the skyrmion structure in this system. Specifically, the extent of skyrmion lattice phase is strongly enhanced when stress is applied perpendicular to magnetic field, and this extent is gradually reduced under stresses parallel to the field. So far, the microscopic mechanism for the stress effect remains vague, and urgently deserves to be uncovered in order to understand the physics and even speed up the application process^23^.

Fortunately, some spin models were proposed and the ordered phases found in experiments on bulk MnSi have been successfully reproduced, allowing one to explore the stress effect based on such models^12^. Usually, uniaxial stresses may lead to lattice distortion, and in turn modulate exchange anisotropies in a magnetic system^24, 25^. For example, the exchange anisotropy has been proven to be very important in strained manganite thin films^26, 27^ and in strained iron pnictides^28, 29^. Furthermore, the DM interaction in chiral magnets along the compressive axis is found to be largely enhanced when a pressure is applied, as revealed in earlier experiments (on FeGe thin films)^25^ and first-principles calculations (on Mn_1−*x*_Fe_*x*_Ge)^30^. Thus, it is essential to clarify the role of interaction anisotropy in modulating the magnetic orders in order to understand the strain effect in chiral magnets. More importantly, such a study may provide useful information about magnetic ordering in similar magnets with anisotropic interactions.

In this work, we study the classical Heisenberg spin model including anisotropic FM exchange and DM interactions on a three-dimensional lattice by combining variational zero-*T* calculations with Monte Carlo (MC) simulations to understand the stress induced magnetic orders in bulk MnSi. The experimentally reported reorientation of wave vector of the helical order and the stability of skyrmion lattice phase in experimentally determined phase diagrams under uniaxial stress are qualitatively reproduced when the interaction anisotropies are taken into account.

## Model and Methods

In this work, the classical Heisenberg spin model taking account of the DM interaction and anisotropic exchange applicable to strained MnSi is considered and its Hamiltonian is given by:

1 $$\begin{array}{rcl}H & = & -\sum _{{\bf{i}}}({J}_{x}{{\bf{S}}}_{{\bf{i}}}\cdot {{\bf{S}}}_{{\bf{i}}+\hat{x}}+{J}_{y}{{\bf{S}}}_{{\bf{i}}}\cdot {{\bf{S}}}_{{\bf{i}}+\hat{y}}+{J}_{z}{{\bf{S}}}_{{\bf{i}}}\cdot {{\bf{S}}}_{{\bf{i}}+\hat{z}})\\  &  & -\sum _{{\bf{i}}}({D}_{x}{{\bf{S}}}_{{\bf{i}}}\times {{\bf{S}}}_{{\bf{i}}+\hat{x}}\cdot \hat{x}+{D}_{y}{{\bf{S}}}_{{\bf{i}}}\times {{\bf{S}}}_{{\bf{i}}+\hat{y}}\cdot \hat{y}+{D}_{z}{{\bf{S}}}_{{\bf{i}}}\times {{\bf{S}}}_{{\bf{i}}+\hat{z}}\cdot \hat{z})-h\sum _{{\bf{i}}}{{\bf{S}}}_{{\bf{i}}}^{z},\end{array}$$

where **S**_**i**_ represents the Heisenberg spin with unit length on site **i**, ($\hat{x}$, $\hat{y}$, $\hat{z}$) are the basis vectors of the cubic lattice considered here. The first term is the anisotropic FM exchange between the nearest neighbors with interaction constant *J*_*μ*_ (*μ* = *x*, *y*, *z*). The second term is the anisotropic DM interaction with prefactor *D*_*μ*_ (*μ* = *x*, *y*, *z*). The last term is the Zeeman coupling with magnetic field *h* along the [001] direction. For simplicity, *J*_*x*_, *J*_*y*_, the lattice constant, and the Boltzmann constant are set to unity. In this work, the ground states are obtained using an analytical approach, and the finite-*T* phase diagrams are estimated by MC simulations. It is noted that the system size studied in this work is much larger than that of skyrmion, and the demagnetization energies which are important in nanostructures^31, 32^ can be safely ignored with respect to the DM interaction and FM exchange^33^.

In the isotropic bulk system under zero *h*, the ground state is a helical order with wave vector^8^ **k** = arctan(*D*/$\sqrt{3}$ *J*) (1, 1, 1) and its orientation is usually related to the weak magneto-crystalline anisotropy^34, 35^. Furthermore, uniaxial anisotropy also can efficiently modulate the magnetic states in chiral magnets^36, 37^ and other magnetic materials^38, 39^. However, an exact solution of the model further considering the magneto-crystalline anisotropy is hard to access using the variational method. Thus, such an anisotropy is not considered here in order to help one to understand the effect of interaction anisotropy, and our physical conclusions are not affected by this ignorance. Interestingly, when an interaction anisotropy is considered, the ground-state is still a single-**k** helical order with **k = **(*k*_*x*_, *k*_*y*_, *k*_*z*_), to be explained latter. Without loss of generality, we set the rotation axis vector **R** and initial spin **S**_**0**_, respectively, to be:

2 $$\begin{array}{c}{\bf{R}}=(\sin \,\phi \,\cos \,\theta ,\,\sin \,\phi \,\sin \,\theta ,\,\cos \,\phi )\\ {{\bf{S}}}_{{\bf{0}}}=(\cos \,\phi \,\cos \,\theta ,\,\cos \,\phi \,\sin \,\theta ,-\sin \,\phi ).\end{array}$$

Then, the spin vector **S**_**i**_, the energy per site *E*, and the effective field ***f***_**i**_, can be calculated respectively by:

3 $${{\bf{S}}}_{{\bf{i}}}=\,\sin ({\bf{k}}\cdot {\bf{i}})\cdot {\bf{R}}\times {{\bf{S}}}_{{\bf{0}}}+\,\cos ({\bf{k}}\cdot {\bf{i}})\cdot {{\bf{S}}}_{{\bf{0}}}$$

4 $$E=-({J}_{x}\,\cos \,{k}_{x}+{J}_{y}\,\cos \,{k}_{y}+{J}_{z}\,\cos \,{k}_{z})-({D}_{x}\,\sin \,\phi \,\cos \,\theta \,\sin \,{k}_{x}+{D}_{y}\,\sin \,\phi \,\sin \,\theta \,\sin \,{k}_{y}+{D}_{z}\,\cos \,\phi \,\sin \,{k}_{z}),$$

and

5 $${f}_{{\bf{i}}}=-\frac{\delta {\boldsymbol{H}}}{\delta {{\bf{S}}}_{{\bf{i}}}}=\sum _{\mu }{{\boldsymbol{J}}}_{\mu }{{\bf{S}}}_{{\bf{i}}+\hat{\mu }}+\sum _{\mu }{{\boldsymbol{D}}}_{\mu }{{\bf{S}}}_{{\bf{i}}+\hat{\mu }}\times \hat{\mu }.$$

By optimizing for **k** and (*θ*, *φ*), we obtain the following set of equations:

6 $$\{\begin{array}{l}{J}_{x}\,\tan \,{k}_{x}={D}_{x}\,\sin \,\phi \,\cos \,\theta \\ {J}_{y}\,\tan \,{k}_{y}={D}_{y}\,\sin \,\phi \,\sin \,\theta \\ {J}_{z}\,\tan \,{k}_{z}={D}_{z}\,\cos \,\phi \\ {D}_{z}\,\sin \,{k}_{z}\,\tan \,\phi ={D}_{x}\,\cos \,\theta \,\sin \,{k}_{x}+{D}_{y}\,\sin \,\theta \,\sin \,{k}_{y}\\ {D}_{x}\,\sin \,\phi \,\sin \,\theta \,\sin \,{k}_{x}={D}_{y}\,\sin \,\phi \,\cos \,\theta \,\sin \,{k}_{y}\end{array}$$

Here, the last two equations ensure **S**_**i**_ × ***f***_**i**_ = 0, confirming that the single-**k** helical order is the ground state. Then, we can uncover the ground-state of the system at zero *h* for given *D*_*μ*_ and *J*_*μ*_.

In addition, the finite-*T* phase diagram under various *h* is also calculated by MC simulations. Following earlier work^12^, a compensation term is considered in the model Hamiltonian to minimize the discretization errors in the simulations, which can be given by:

7 $$\begin{array}{rcl}{H}_{C} & = & \frac{1}{16}\sum _{{\bf{i}}}({J}_{x}{{\bf{S}}}_{{\bf{i}}}\cdot {{\bf{S}}}_{{\bf{i}}+2\hat{x}}+{J}_{y}{{\bf{S}}}_{{\bf{i}}}\cdot {{\bf{S}}}_{{\bf{i}}+2\hat{y}}+{J}_{z}{{\bf{S}}}_{{\bf{i}}}\cdot {{\bf{S}}}_{{\bf{i}}+2\hat{z}})\\  &  & +\,\frac{1}{8}\sum _{{\bf{i}}}({D}_{x}{{\bf{S}}}_{{\bf{i}}}\times {{\bf{S}}}_{{\bf{i}}+2\hat{x}}\cdot \hat{x}+{D}_{y}{{\bf{S}}}_{{\bf{i}}}\times {{\bf{S}}}_{{\bf{i}}+2\hat{y}}\cdot \hat{y}+{D}_{z}{{\bf{S}}}_{{\bf{i}}}\times {{\bf{S}}}_{{\bf{i}}+2\hat{z}}\cdot \hat{z}).\end{array}$$

The simulation is performed on an *N* = 24^3^ cubic lattice with period boundary conditions using the standard Metropolis algorithm^40^ and the parallel tempering algorithm^41^. We take an exchange sampling after every 10 standard MC steps. Typically, the initial 6 × 10^5^ steps are discarded for the equilibrium consideration and additional 6 × 10^5^ steps are retained for the statistic averaging of simulation. Occasional checks are made on a larger lattice of up to *N* = 40^3^ to ensure that the finite-size effect never affects our conclusion. We characterize the spin structures by performing the Fourier transform

8 $$\langle {{\bf{S}}}_{{\bf{k}}}\rangle =\frac{1}{N}\sum _{{\bf{i}}}\langle {{\bf{S}}}_{{\bf{i}}}\rangle {e}^{-i{\bf{k}}\cdot {\bf{i}}},$$

and then calculating the intensity profile |〈**S**_**k**_〉|^2^. Furthermore, we also calculate the longitudinal susceptibility *χ*_*z*_, and the uniform chirality *χ*

9 $$\chi =\frac{1}{8\pi }\sum _{{\bf{i}}}[{{\bf{S}}}_{{\bf{i}}}\cdot ({{\bf{S}}}_{{\bf{i}}+\hat{x}}\times {{\bf{S}}}_{{\bf{i}}+\hat{y}})+{{\bf{S}}}_{{\bf{i}}}\cdot ({{\bf{S}}}_{{\bf{i}}-\hat{x}}\times {{\bf{S}}}_{{\bf{i}}-\hat{y}})].$$

to estimate the phase transition points^8^.

## Results and Discussion

### Wave vector reorientation of helix

First, we study the cases where the FM exchange and DM interaction anisotropy have the same magnitude at zero *h*. Generally, one may define an anisotropy magnitude *α* and a ratio of the DM interaction to the exchange interaction *β*:

10 $$\alpha =\frac{{J}_{x,y}}{{J}_{z}},\beta =\frac{{D}_{\mu }}{{J}_{\mu }}.$$

Here, Eq. (4) is updated to:

11 $$\begin{array}{rcl}E & = & -{J}_{x}[(\cos \,{k}_{x}+\,\cos \,{k}_{y}+\frac{1}{\alpha }\,\cos \,{k}_{z})+(\beta \,\sin \,\phi (\cos \,\theta \,\sin \,{k}_{x}+\,\sin \,\theta \,\sin \,{k}_{y})\\  &  & +\,\frac{\beta }{\alpha }\,\cos \,\phi \,\sin \,{k}_{z})].\end{array}$$

Once the energy expression is minimized, we obtain the modulus of wave vector **k** and energies *E* in several specific cases:

(1) helical spin state with **k =** *k*(0, 0, 1)

12 $$\begin{array}{rcl}{k}_{z} & = & \arctan (\beta )\,{\rm{for}}\,\phi \,=\,0,\\ {E}_{[001]} & = & -{J}_{x}(2+\frac{\sqrt{1+{\beta }^{2}}}{\alpha }).\end{array}$$

(2) helical spin state with **k** = *k*(1, 1, 0)

13 $$\begin{array}{rcl}{k}_{x,y} & = & \arctan (\frac{\beta }{\sqrt{2}})\,\,{\rm{for}}\,\theta =\frac{\pi }{4},\phi \,=\,\frac{\pi }{2},\\ {E}_{[110]} & = & -{J}_{x}(\sqrt{4+2{\beta }^{2}}+\frac{1}{\alpha }).\end{array}$$

(3) helical spin state with **k** = (*k*_*x*_, *k*_*y*_, *k*_*z*_)

14 $$\begin{array}{rcl}{k}_{x,y} & = & \arcsin \sqrt{\frac{{\alpha }^{2}(1+{\beta }^{2})-1}{{\alpha }^{2}({\beta }^{2}+3)}}\,,\,{k}_{z}=\arcsin \sqrt{\frac{{\beta }^{2}-2{\alpha }^{2}+2}{{\beta }^{2}+3}}\\ {\rm{for}}\,\phi  & = & \sqrt{\frac{2{\alpha }^{2}(1+{\beta }^{2})-2}{{\beta }^{2}(2{\alpha }^{2}+1)}},\theta =\frac{\pi }{4},\\ {E}_{[xxz]} & = & -{J}_{x}(\frac{3+{\beta }^{2}}{\alpha }\sqrt{\frac{2{\alpha }^{2}+1}{{\beta }^{2}+3}}).\end{array}$$

Furthermore, it should be mentioned that the [*xxz*] helical state is accessed only in the region defined by 0 < *φ* < π/2 and

15 $$\sqrt{\frac{1}{1+{\beta }^{2}}} < \alpha  < \sqrt{\frac{2+{\beta }^{2}}{2}}.$$

**Figure 1:**
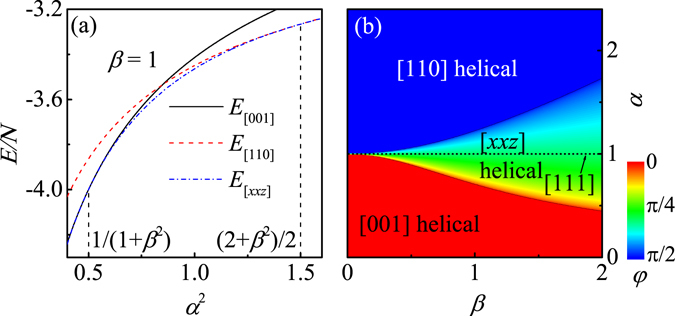
(**a**) The local energies as a function of *α*. (**b**) The ground-state phase diagram in the space of (*α*, *β*).

It is expected that *α* increases (*α* > 1) when a compressive strain is applied along the [110] axis. Interestingly, the [110] helical order will win out over the [111] helical phase for *α* > $\sqrt{1.5}$, as clearly shown in Fig. 1(a) which gives the calculated energies for a fixed *β* = 1. Thus, the stress-induced reorientation of the wave vector of the helix observed in experiments can be qualitatively reproduced in our anisotropic model. Similarly, the [111] helical order will be replaced by the [001] one for small *α* < $\sqrt{2}/2$, related to the case of compressive (tensile) stress applied along the [001] ([110]) axis, to some extent^42, 43^. The calculated ground-state phase diagram in the (*α*, *β*) parameter plane is shown in Fig. 1(b) which can be divided into three parameter regions with different helical orders. It is noted that the helical propagation direction gradually turns to the stress axis (*φ* gradually changes) when the anisotropy magnitude is increased, well consistent with experimental observations. Furthermore, the *α*-region favoring the [*xxz*] helical order is seriously suppressed as *β* decreases, demonstrating that the helical order in chiral magnet with a weak DM interaction can be easily modulated by uniaxial stress^44^.

**Figure 2:**
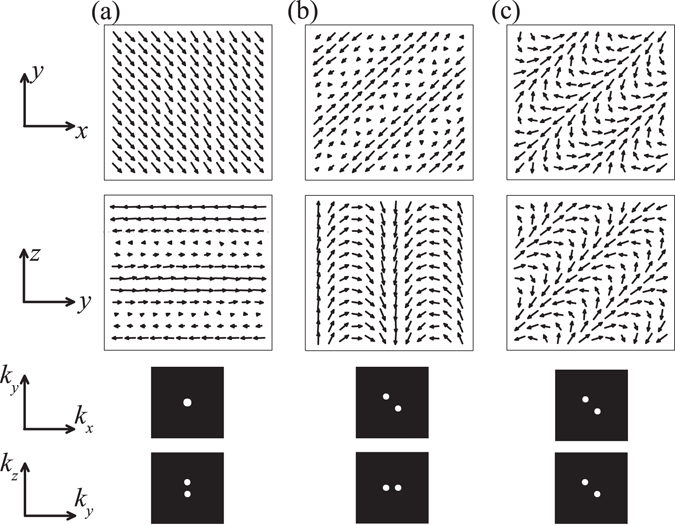
A plot of the spin configurations projected on the *xy* plane (up) and projected on the *yz* plane (middle). At the bottom of each figure are the plots of the Bragg intensity from Fourier transition which shows the sets of helix vectors. The parameters are (**a**) (*α*, *β*) = (0.866, 0.577), (**b**) (*α*, *β*) = (1.155, 0.816), and (**c**) (*α*, *β*) = (1, 1) at *T* = 0.01.

As a matter of fact, these helical spin orders are also confirmed in our MC simulations. For example, the [001] helical order is stabilized at low *T* for (*α*, *β*) = (0.866, 0.577), and its spin configuration and the Bragg intensity are shown in Fig. 2(a). In one in-plane (*xy*) lattice layer, all the spins are parallel with each other. In addition, the spins of the chain along the [001] direction form a spiral structure, clearly demonstrating the helical order with the wave vector **k** = (0, 0, *k*). For *α* < 1 (i.e. compressive strain applied along the [001] axis), the exchange interaction *J*_*z*_ and DM interaction *D*_*z*_ play an essential role in determining the ground-state, and their competition results in the appearance of the [001] helical order. Thus, the compressive strain will tune the wave vector from the [111] axis to the stress axis, as reported in experiments. Similarly, the [110] helical order (Fig. 2(b)) and the [111] helical order (Fig. 2(c)) can be developed for (*α*, *β*) = (1.155, 0.816) and (*α*, *β*) = (1, 1), respectively. Furthermore, these spin orders can be also reflected in the calculated Bragg intensities, as given in the bottom of Fig. 2.

On the other hand, it is noted that the exchange anisotropy may not have the same magnitude as the DM interaction anisotropy, especially in the systems where the spin-orbit coupling is anisotropic^24^. Without losing the generality, we also investigate these effects. We define the following two parameters:

16 $$\gamma =\frac{{D}_{x,y}}{{J}_{x,y}},\xi =\frac{{D}_{z}}{{J}_{z}}.$$

Following earlier work^24^, one has a constraint $\frac{{J}_{z}}{{J}_{x,y}}=\sqrt{\frac{(1+{\gamma }^{2})}{(1+{\xi }^{2})}}$, and Eq. (4) is updated to:

17 $$\begin{array}{rcl}E & = & -{J}_{x}[(\cos \,{k}_{x}+\,\cos \,{k}_{y}+\sqrt{\frac{(1+{\gamma }^{2})}{(1+{\xi }^{2})}}\cos \,{k}_{z})\\  &  & -(\gamma \,\sin \,\phi (\cos \,\theta \,\sin \,{k}_{x}+\,\sin \,\theta \,\sin \,{k}_{y})+\sqrt{\frac{(1+{\gamma }^{2})}{(1+{\xi }^{2})}}\xi \,\cos \,\phi \,\sin \,{k}_{z})].\end{array}$$

**Figure 3:**
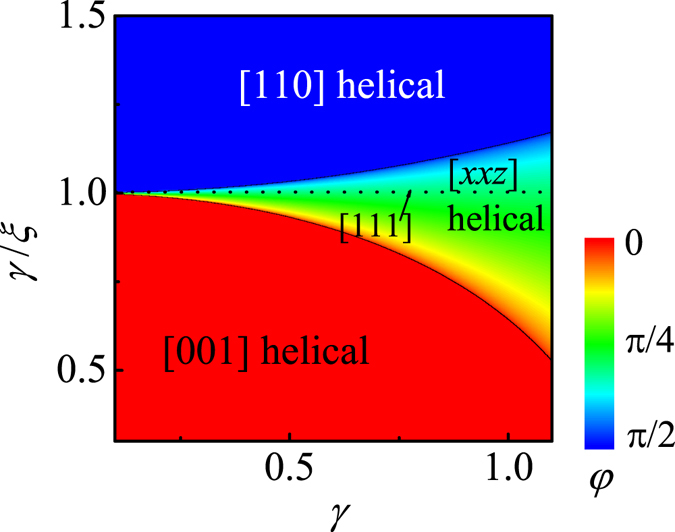
The ground-state phase diagram in the space of (*γ*, *γ*/*ξ*).

Similarly, the phase boundaries in the phase diagram can be rigorously obtained by comparing these energies of the helical orders, and the calculated ground-state phase diagram in the (*γ*, *γ*/*ξ*) parameter plane is shown in Fig. 3. It is clearly demonstrated that the helical order can be effectively modulated by these parameters, further validating the conclusion that the interaction anisotropy may be important in understanding the uniaxial stress dependence of ground-state in chiral magnets such as MnSi.

### Effect of stress on skyrmion lattice phase

With a magnetic field applied along the [001] direction, the skyrmion lattice phase appears in a small *h*-*T* region. A transverse (longitudinal) pressure further stabilizes (destabilizes) the skyrmion lattice phase, resulting in the expanding (shrinking) of this *T*-region with this phase, as reported in earlier experiments on bulk MnSi^21, 22^. This behavior is also captured in the present anisotropic spin model.

**Figure 4:**
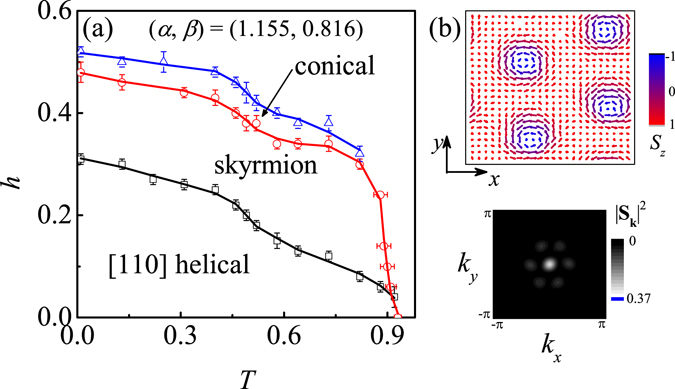
(**a**) The estimated phase diagram in the (*T*, *h*) plane for (*α*, *β*) = (1.155, 0.816), and (**b**) A plot of the in-plane layer spin configuration for the tube skyrmion phase. The intensity profile is also given in the bottom of (**b**).

Figure 4(a) shows the simulated phase diagram for (*α*, *β*) = (1.155, 0.816). Even with the compensation term, the skyrmion lattice phase remains stable at low *T*, demonstrating the prominent role of the interaction anisotropies in modulating the skyrmion lattice phase. This phenomenon can be understood by analyzing the spin structures. For one spin chain along the *z* direction in the [110] helical order, all the spins are parallel with each other, and *J*_*z*_ interaction is completely satisfied. Thus, there is no energy loss from the *J*_*z*_ interaction due to the transition from the helical order to the tube skyrmion phase, resulting in the expansion of the *T*-region for the skyrmion lattice phase. In some extent, this behavior is similar to that of the two-dimensional system in which a rather large *T*-region for the skyrmion lattice phase has been reported both experimentally^4, 5^ and theoretically^45^ as a result of the suppression of the competing conical phase. In Fig. 4(b), we show a snapshot (one in-plane lattice layer) of the skyrmion lattice phase and the Bragg intensity at *T* = 0.07 and *h* = 0.46. The skyrmion phase with the hexagonal symmetry is clearly confirmed. It is noted that the anisotropy magnitude may not be so large in real materials, and the skyrmion lattice phase at *T* → 0 predicted here has not been reported experimentally. However, this work indeed manifests the important role of the interaction anisotropy in modulating the skyrmions.

**Figure 5:**
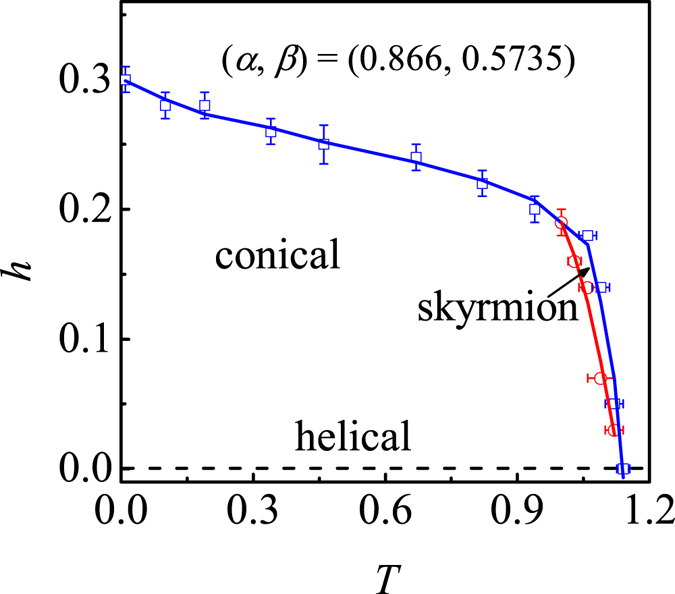
The estimated phase diagram in the (*T*, *h*) plane for (*α*, *β*) = (0.866, 0.577).

On the contrary, the stability of the skyrmion lattice phase is significantly suppressed for *α* < 1, as shown in Fig. 5 which gives the phase diagram for (*α*, *β*) = (0.866, 0.5735). With the increase of *J*_z_ (*α* decreases), the energy loss from the *J*_z_ interaction due to the transition to the skyrmion lattice phase increases, resulting in the destabilization of the skyrmion lattice phase. As a matter of fact, earlier experiment revealed that an in-plane tensile strain destabilizes the skyrmion lattice phase^37^, consistent with our simulations. Furthermore, it is clearly shown that the helical order is only stabilized at zero *h*, which can be explained analytically. The spins in an in-plane layer are parallel with each other in the [001] helical order, exhibiting a quasi-one-dimensional property. In this case, the energy of the conical phase under small *h* can be written by

18 $${E}_{{\rm{con}}}=-{D}_{z}\,\sin \,{k}_{z}\sqrt{1-\frac{2\,{\cos }^{2}\varphi }{1+\,\cos \,{k}_{z}}}-{J}_{z}\,\cos \,{k}_{z}-h\,\cos \,\varphi ,$$

where *ϕ* is the cone half-angle (for the [001] helical order, *ϕ* = π/2). Once the energy term is minimized, we obtain

19 $$h(1+\,\cos \,{k}_{z})\sqrt{1-\frac{2{\cos }^{2}\varphi }{1+\,\cos \,{k}_{z}}}=2{D}_{z}\,\sin \,{k}_{z}\,\cos \,\varphi .$$

Thus, it is clearly indicated that the [001] helical order can only be developed at zero *h*. In fact, earlier experiments have revealed that both the helical order and skyrmion lattice phase can be destabilized by the longitudinal pressure^21^. Here, our work suggests that the conical phase will completely replace the helical one at finite *h* in those systems with strong interaction anisotropies.

## Conclusion

In conclusion, we have studied the uniaxial stress effects on the magnetic orders of bulk MnSi based on the spatially anisotropic spin model. Several experimental observations are qualitatively reproduced by the analytical calculation and Monte Carlo simulations of the model. It is suggested that the helical orders as well as the skyrmion lattice phase can be effectively modulated by the interaction anisotropy tuned by the applied pressure, especially for the system with a weak DM interaction. The present work may provide new insights into the understanding of the magnetic orders in the strained MnSi.
